# The causal links between gut microbiota and both acute and chronic renal failure: A two-sample mendelian randomization study

**DOI:** 10.1097/MD.0000000000046336

**Published:** 2025-12-12

**Authors:** Wenhua Yang

**Affiliations:** a Dialysis Room, People’s Hospital of Linquan County, Linquan, China.

**Keywords:** acute renal failure, chronic renal failure, genome-wide association study, gut microbiota, mendelian randomization

## Abstract

This study aimed to investigate the potential causal relationships between gut microbiota composition and both acute renal failure (ARF) and chronic renal failure (CRF), using a two-sample Mendelian randomization approach. We characterized the gut microbiota composition at various taxonomic levels (phylum, class, order, family, and genus) and obtained genome-wide association study data for both ARF and CRF from the FinnGen and UK Biobank databases. Subsequently, we employed a two-sample Mendelian randomization approach to explore the causal relationship between the gut microbiota and renal failure. We identified 10 causal relationships between the genetic liability in the gut microbiome and ARF. We found 4 associations between genetic liability in the gut microbiome and CRF. The results indicated that *Haemophilus* (inverse variance weighted, IVW OR = 0.816, 95% CI = 0.719–0.926, *P* = .002, q = 0.054) and *Ruminococcaceae* UCG005 (IVW OR = 0.804, 95% CI = 0.694–0.931, *P* = .004, q = 0.078) showed negative associations with ARF. Moreover, *Ruminococcaceae* UCG005 (IVW OR = 1.001, 95% CI = 1.00–1.002, *P* = .008, q = 0.091) positively correlated with CRF risk. Sensitivity analyses did not provide statistical evidence of bias due to pleiotropy or genetic confounding factors. Our research contributes significantly to the growing body of knowledge linking the gut microbiota to renal health, offering new perspectives for the diagnosis, treatment, and prevention of renal diseases.

## 1. Introduction

Gut microbiota plays a crucial role in maintaining human health through various mechanisms, including immune modulation, nutrient absorption, and metabolic regulation.^[1]^ Recent studies have highlighted the potential link between gut microbiota composition and renal diseases.^[2,3]^ Dysbiosis of the gut microbiome has been associated with both acute renal failure (ARF) and chronic renal failure (CRF), suggesting that alterations in microbial communities may contribute to the pathogenesis of these conditions.^[4,5]^ For instance, patients with CRF exhibit distinct gut microbiota profiles compared to healthy individuals, characterized by reduced diversity and overrepresentation of certain bacterial taxa.^[6]^ Similarly, ARF has been linked to dysregulated gut microbiota, although specific microbial signatures remain less well-defined.^[7]^

Modulation of the gut microbiota offers promising therapeutic strategies for preventing and treating renal diseases.^[8]^ Preclinical studies have demonstrated that interventions such as probiotics, prebiotics, and fecal microbiota transplantation can ameliorate renal dysfunction by restoring the microbial balance and reducing systemic inflammation.^[9]^ In particular, probiotics have shown efficacy in mitigating uremic toxins and improving kidney function in animal models of CRF.^[10]^ Furthermore, targeting the gut-kidney axis through dietary modifications or pharmacological agents could potentially slow disease progression and improve patient outcomes.^[11]^ These findings underscore the need for further research into the mechanisms underlying gut-kidney crosstalk and how it can be harnessed for clinical benefits.

Gut microbiota dysbiosis can lead to various diseases, including those affecting the renal health. An imbalance in microbial communities can result in the production of harmful metabolites, such as indoxyl sulfate and p-cresyl sulfate, which are uremic toxins that contribute to renal damage.^[12]^ Moreover, the gut-kidney axis involves complex signaling pathways, including the renin-angiotensin system^[13]^ and toll-like receptor signaling,^[14]^ which mediate the inflammatory response and injury in renal tissues. Understanding these mechanisms is critical for developing targeted therapies aimed at modulating gut microbiota to protect renal function.

This study aimed to investigate the causal relationships between gut microbiota composition and both ARF and CRF, using a two-sample Mendelian randomization (MR) approach. By leveraging genome‐wide association study (GWAS) data from large consortia, such as FinnGen and UK Biobank, we sought to identify genetic variants associated with gut microbiota that may influence renal health. Our research provides novel insights into the gut-kidney axis and highlights potential therapeutic targets for renal diseases. Unlike previous studies that primarily focused on observational associations, our MR analysis allowed for causal inference, thereby strengthening the evidence base for the role of gut microbiota in renal health. This study represents a significant advancement in understanding the interactions between the gut microbiome and renal function, paving the way for innovative preventive and therapeutic strategies.

## 2. Materials and methods

### 2.1. Ethical compliance and data governance

This study adhered to the ethical guidelines outlined in the Declaration of Helsinki and followed the STROBE-MR reporting standards for transparency in MR research.^[15]^ Summary-level data were derived from publicly accessible GWASs approved by institutional review boards.

All original studies obtained informed consent from participants, and no additional ethical approval was required for this secondary analysis of anonymized data.

### 2.2. Exposure data

SNPs associated with the composition of the human gut microbiome were chosen as instrumental variables (IVs) from a GWAS dataset of the international MiBioGen consortium.^[16]^ This large-scale, multi-ethnic GWAS integrated 16S ribosomal RNA gene sequencing profiles and genotyping data. The data came from 18,340 participants in 24 cohorts across multiple countries including the USA, Canada, Israel, South Korea, Germany, Denmark, the Netherlands, Belgium, Sweden, Finland, and the UK. The aim was to investigate the relationship between autosomal human genetic variants and the gut microbiome. In total, 211 taxa, which included 131 genera, 35 families, 20 orders, 16 classes, and 9 phyla, were incorporated.^[16]^

### 2.3. Outcome data

ARF data (N = 6429 cases; 396,706 controls) (https://storage.googleapis.com/finngen-public-data-r10/summary_stats/finngen_R10_N14_ACUTERENFAIL.gz) was sourced from FinnGen R10. The CRF data (N = 31,445 participants) (https://broad-ukb-sumstats-us-east-1.s3.amazonaws.com/round2/additive-tsvs/N18.gwas.imputed_v3.both_sexes.tsv.bgz) were obtained from the UK Biobank.

### 2.4. Instrumental variable (IV) selection and quality control

To explore the causal relationship between the gut microbiome and renal failure risk, bacterial taxa were analyzed at 5 hierarchical levels (phylum, class, order, family, genus), with each taxon regarded as a characteristic feature. A series of quality-control steps were taken to select appropriate IVs.

SNPs significantly associated with the gut microbiome were chosen as IVs using 2 thresholds: a genome – wide significance level of 5 × 10^−8^ and a locus – wide level of 1 × 10^−6^. The latter was used due to the limited number of SNPs obtained from the former. The minor allele frequency threshold for variants was set at 0.01.

A clumping procedure (*R*^2^ < 0.01, 10,000 kb) was performed to assess linkage disequilibrium among SNPs, as linkage disequilibrium can skew MR results. Palindromic, ambiguous, and duplicated SNPs were removed during allele harmonization to align with the human genome reference (build 37).

MR-PRESSO and MR-Egger regression tests were used to detect horizontal pleiotropy. SNPs were removed iteratively based on MR-PRESSO outlier test *P* values until the global test *P* value was non-significant (*P* > .05). The remaining SNPs were used for subsequent MR analysis.

### 2.5. Mendelian randomization analysis

We conducted a MR analysis to explore the causal relationship between microbiome features and renal failure. For features with a single instrumental variable (IV), the Wald ratio test estimated the association with renal failure.^[17]^ For features with multiple IVs, we used 5 MR methods: inverse-variance weighted (IVW) test,^[18]^ weighted mode,^[19]^ MR-Egger regression,^[20]^ weighted median estimator,^[21]^ and Simple mode. The IVW method, being more powerful under certain conditions, formed the basis of our main results for multi-IV features, with the other four as complements.

We set a multiple-testing significance threshold (*P* < .05/n, n = effective number of independent taxa) at each taxonomic level (phylum, class, order, family, genus). To assess result robustness, we performed a leave-one-out analysis to check for single-SNP influence. Comparing the variance explained by IVs for exposure and outcome helped establish directional credibility. F statistics were calculated to detect weak instrument bias, and IVs with F < 10 were excluded.

All statistical analyses were performed using the R packages “TwoSampleMR (version 0.6.7)^[22]^” and “MR-PRESSO (version 1.0).^[23]^”

### 2.6. Heterogeneity

We assessed heterogeneity among instrumental variables using Cochran’s Q statistics with the “TwoSampleMR” package. A Q greater than the number of instruments minus one or a Q statistics *P* value < .05 indicates heterogeneity, which may imply invalid instruments. This analysis is crucial for ensuring the validity of our Mendelian randomization results.

## 3. Results

**Figure 1.**
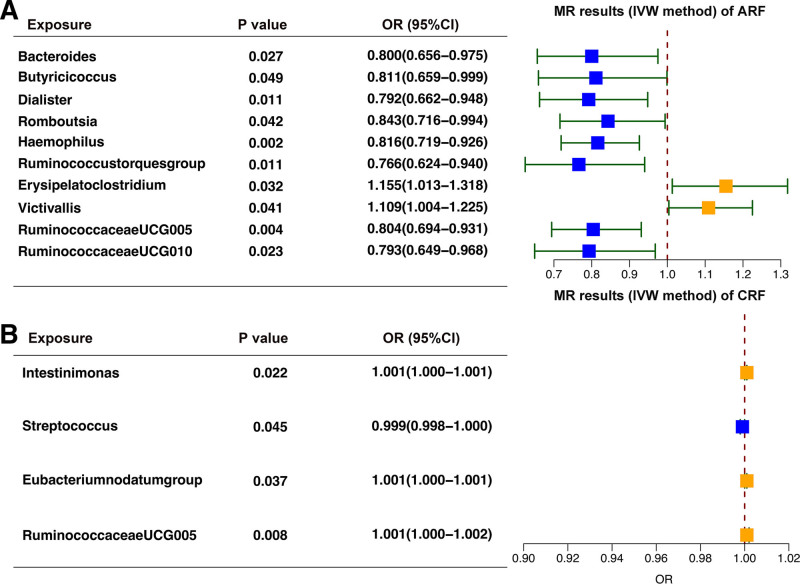
Forest plot of IVW analysis results of the effect of gut microbiota on ARF (A) and CRF (B). ARF = acute renal failure, CRF = chronic renal failure, IVW = inverse variance weighted.

Table 1 and Figure 1 present the findings, indicating that ten bacterial genera, namely *Bacteroides, Butyricicoccus, Dialister, Erysipelatoclostridium, Haemophilus, Romboutsia, Ruminococcaceae* UCG005*, Ruminococcaceae* UCG010*, Ruminococcustorques group*, and *Victivallis*, had *P* values below 0.05 in the IVW analysis. IVW analysis revealed that *Erysipelatoclostridium* (odds ratio [OR] = 1.155, 95% confidence interval [95% CI] = 1.013–1.318, *P* = .032) and *Victivallis* (OR = 1.109, 95% CI = 1.004–1.225, *P* = .041) had a deleterious effect on ARF, whereas other bacterial genera showed a protective role against ARF (Table 1; Fig. 1).

**Table 1:** Mendelian randomization analysis results between gut microbiota and ARF/CRF at genus levels.

Gut microbiota	Trait	MR analysis												
		Nsnp	Beta	OR (95% CI)	Methods			Heterogeneity		Horizontal pleiotropy			MR-PRESSO	
						*P* value	FDR	Cochran’s Q	*P* value	Egger intercept	SE	*P* value	RSSobs	*P* value
Bacteroides	ARF	12	−0.223	0.8 (0.656, 0.975)	Inverse variance weighted	.027	0.501							
			−0.077	0.926 (0.71, 1.207)	Weighted mode	.806	0.973							
			0.282	1.326 (0.465, 3.781)	Weighted median	.575	0.989	9.611	.566	−0.033	0.035	.358	11.486	.566
			−0.057	0.944 (0.649, 1.375)	Simple mode	.790	0.993							
			−0.050	0.951 (0.65, 1.391)	MR Egger	.609	0.995							
Butyricicoccus		9	−0.209	0.811 (0.659, 0.999)	Inverse variance weighted	.049	0.501							
			−0.096	0.909 (0.597, 1.383)	Weighted mode	.376	0.973							
			−0.153	0.858 (0.653, 1.127)	Weighted median	.284	0.989	6.639	.576	−0.011	0.018	.561	7.821	.656
			−0.157	0.855 (0.6, 1.218)	Simple mode	.403	0.993							
			−0.149	0.862 (0.618, 1.203)	MR Egger	.668	0.995							
Dialister		12	−0.233	0.792 (0.662, 0.948)	Inverse variance weighted	.011	0.330							
			−0.198	0.82 (0.368, 1.828)	Weighted mode	.150	0.973							
			−0.246	0.782 (0.611, 1)	Weighted median	.041	0.989	12.389	.335	−0.003	0.030	.932	14.820	.347
			−0.366	0.693 (0.451, 1.067)	Simple mode	.129	0.993							
			−0.346	0.708 (0.466, 1.075)	MR Egger	.638	0.995							
Erysipelatoclostridium		16	0.145	1.155 (1.013, 1.318)	Inverse variance weighted	.032	0.501							
			0.074	1.076 (0.622, 1.863)	Weighted mode	.592	0.973							
			0.115	1.122 (0.935, 1.348)	Weighted median	.209	0.989	15.650	.406	0.006	0.023	.798	17.851	.387
			0.098	1.103 (0.779, 1.561)	Simple mode	.607	0.993							
			0.094	1.099 (0.762, 1.585)	MR Egger	.796	0.995							
Haemophilus		13	−0.203	0.816 (0.719, 0.926)	Inverse variance weighted	.002	0.054							
			−0.263	0.769 (0.564, 1.047)	Weighted mode	.139	0.973							
			−0.192	0.825 (0.701, 0.971)	Weighted median	.024	0.989	5.171	.952	0.007	0.017	.684	5.941	.956
			−0.190	0.827 (0.636, 1.075)	Simple mode	.176	0.993							
			−0.188	0.829 (0.652, 1.052)	MR Egger	.123	0.995							
Romboutsia		14	−0.170	0.843 (0.716, 0.994)	Inverse variance weighted	.042	0.501							
			0.136	1.145 (0.717, 1.829)	Weighted mode	.288	0.973							
			−0.149	0.861 (0.682, 1.088)	Weighted median	.192	0.989	10.224	.675	−0.027	0.020	.196	11.995	.665
			−0.220	0.803 (0.561, 1.149)	Simple mode	.302	0.993							
			−0.216	0.806 (0.556, 1.167)	MR Egger	.580	0.995							
RuminococcaceaeUCG005		17	−0.218	0.804 (0.694, 0.931)	Inverse variance weighted	.004	0.078							
			−0.207	0.813 (0.541, 1.222)	Weighted mode	.144	0.973							
			−0.238	0.788 (0.643, 0.966)	Weighted median	.030	0.989	10.986	.810	−0.001	0.017	.954	12.189	.822
			−0.281	0.755 (0.534, 1.069)	Simple mode	.117	0.993							
			−0.237	0.789 (0.583, 1.067)	MR Egger	.335	0.995							
RuminococcaceaeUCG010		8	−0.232	0.793 (0.649, 0.968)	Inverse variance weighted	.023	0.501							
			−0.233	0.792 (0.594, 1.056)	Weighted mode	.299	0.973							
			−0.265	0.767 (0.463, 1.269)	Weighted median	.112	0.989							
			−0.289	0.749 (0.467, 1.202)	Simple mode	.334	0.993	7.029	.426	0.046	0.022	.077	10.734	.400
			−0.849	0.428 (0.234, 0.781)	MR Egger	.033	0.995							
Ruminococcustorquesgroup		13	−0.267	0.766 (0.624, 0.94)	Inverse variance weighted	.011	0.330							
			−0.160	0.852 (0.398, 1.826)	Weighted mode	.595	0.973							
			−0.176	0.839 (0.628, 1.121)	Weighted median	.239	0.989							
			−0.171	0.843 (0.493, 1.443)	Simple mode	.541	0.993	11.885	.455	−0.007	0.024	.780	14.138	.463
			−0.161	0.851 (0.51, 1.422)	MR Egger	.689	0.995							
Victivallis		12	0.104	1.109 (1.004, 1.225)	Inverse variance weighted	.041	0.501							
			0.119	1.126 (0.982, 1.291)	Weighted mode	.447	0.973							
			0.199	1.22 (0.614, 2.424)	Weighted median	.074	0.989							
			0.123	1.131 (0.874, 1.464)	Simple mode	.334	0.993	12.975	.295	−0.013	0.046	.789	15.672	.290
			0.093	1.097 (0.868, 1.387)	MR Egger	.582	0.995							
Eubacteriumnodatumgroup	CRF	11	0.001	1.001 (1, 1.001)	Inverse variance weighted	.037	0.915							
			0.001	1.001 (0.999, 1.003)	Weighted mode	.326	0.976							
			0.001	1.001 (1, 1.001)	Weighted median	.064	0.938	4.558	.919	0.000	0.000	.533	5.655	.921
			0.001	1.001 (1, 1.002)	Simple mode	.294	0.994							
			0.001	1.001 (1, 1.002)	MR Egger	.297	0.965							
Intestinimonas		20	0.001	1.001 (1, 1.001)	Inverse variance weighted	.022	0.915							
			0.000	1 (1, 1.001)	Weighted mode	.820	0.976							
			0.001	1.001 (0.999, 1.002)	Weighted median	.304	0.938							
			0.000	1 (0.999, 1.002)	Simple mode	.755	0.994	12.630	.857	0.000	0.000	.915	14.205	.878
			0.000	1 (0.999, 1.002)	MR Egger	.327	0.965							
RuminococcaceaeUCG005		17	0.001	1.001 (1, 1.002)	Inverse variance weighted	.008	0.091							
			−0.001	0.999 (0.997, 1.001)	Weighted mode	.431	0.976							
			0.001	1.001 (1, 1.002)	Weighted median	.089	0.938							
			0.001	1.001 (1, 1.002)	Simple mode	.427	0.994	16.039	.450	0.000	0.000	.104	18.544	.469
			0.001	1.001 (0.999, 1.003)	MR Egger	.511	0.965							
Streptococcus		17	−0.001	0.999 (0.998, 1)	Inverse variance weighted	.045	0.915							
			0.000	1 (0.997, 1.004)	Weighted mode	.761	0.976							
			0.001	1.001 (0.999, 1.003)	Weighted median	.415	0.938	14.695	.547	0.000	0.000	.443	16.677	.556
			0.000	1 (0.998, 1.002)	Simple mode	.768	0.994							
			0.000	1 (0.998, 1.002)	MR Egger	.813	0.973							

ARF = acute renal failure, CRF = chronic renal failure, FDR = false discovery rate, MR = Mendelian randomization, MR-PRESSO = Mendelian randomization Pleiotropy RESidual Sum and Outlier, RSSobs = Observed Residual Sum of Squares, SE = standard error.

The IVW analysis revealed that *Eubacteriumnodatum* group (OR = 1.001, 95% CI = 1.00–1.001, *P* = .037), *Intestinimonas* (OR = 1.001, 95% CI = 1.00–1.001, *P* = .022), and *Ruminococcaceae* UCG005(OR = 1.001, 95% CI = 1.00–1.002, *P* = .008) had a deleterious effect on CRF, whereas Streptococcus (OR = 0.009, 95% CI = 0.098–1.00, *P* = .045) showed a protective role against CRF (Table 1; Fig. 1).

**Figure 2.**
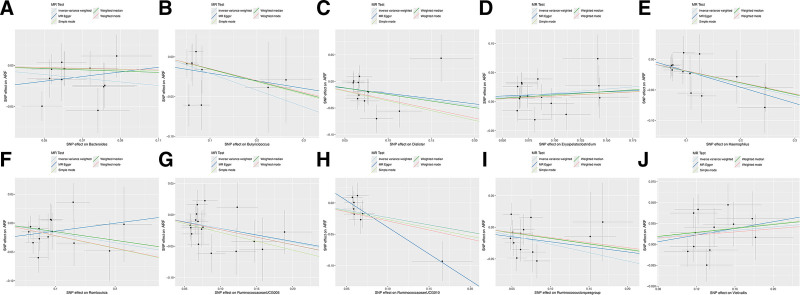
Scatter plots of the MR tests in 10 causal associations of ARF. The slope of the line corresponded to the causal estimation. ARF = acute renal failure, MR = Mendelian randomization.

After false discovery rate correction, we found that *Haemophilus* (IVW OR = 0.816, 95% CI = 0.719–0.926), *P* = .002, q = 0.054), and *Ruminococcaceae* UCG005 (IVW OR = 0.804, 95% CI = 0.694–0.931, *P* = .004, q = 0.078) showed suggestive negative associations with ARF. Moreover, *Ruminococcaceae* UCG005 (IVW OR = 1.001, 95% CI = 1.00–1.002, *P* = .008, q = 0.091) showed suggestive positive associations with CRF (Table 1; Fig. 1). Scatter plots across various tests are displayed in Figures 2 and 4A–D for the ARF and CRF, respectively.

**Figure 3.**
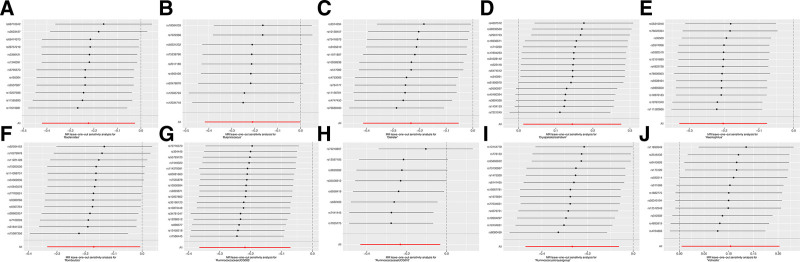
Leave‐one‐out plots for the causal association between gut microbiota and ARF. ARF = acute renal failure.

**Figure 4.**
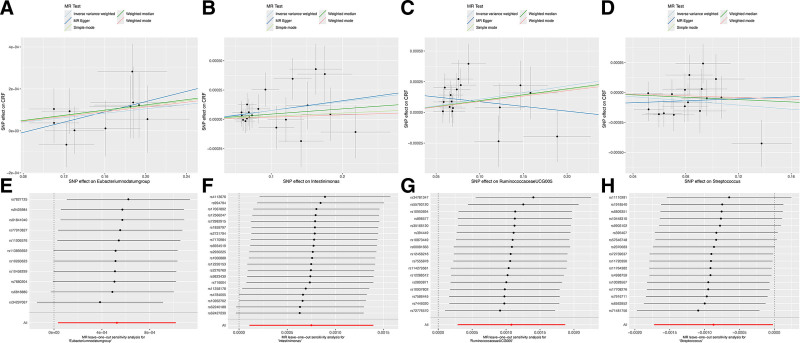
Scatter plots and leave‐one‐out plots for the causal association between gut microbiota and CRF. CRF = chronic renal failure.

In the evaluation of 14 causal relationships, the F-statistics for the IVs ranged from a low of 14.58 to a high of 88.42, suggesting that there was no weak instrument bias present. The findings related to heterogeneity and pleiotropy, detailed in Table 1, did not reach statistical significance (*P* > .05; Table 1). As shown in Figures 3 and 4, the leave-one-out analysis further validated the consistency and robustness of the estimated causal effects (Figs. 3 and 4E–H).

## 4. Discussion

Our study identified several gut microbiota taxa that are associated with ARF and CRF. Specifically, *Bacteroides, Butyricicoccus, Dialister, Erysipelatoclostridium, Haemophilus, Romboutsia, Ruminococcaceae* UCG005, *Ruminococcaceae* UCG010, *Ruminococcus torques*, and *Victivallis* were significantly correlated with ARF. In contrast, *Eubacterium nodatum group, Intestinimonas, Ruminococcaceae* UCG005, and *Streptococcus* have been linked to CRF. After multiple *P* value corrections, *Haemophilus* and *Ruminococcaceae* UCG005 showed protective effects against ARF, using the inverse variance-weighted (IVW) method. Conversely, *Ruminococcaceae* UCG005 promoted CRF progression.

The opposing roles of *Ruminococcaceae* UCG005 in ARF and CRF warrant further investigation. *Ruminococcaceae* UCG005 may exert protective effects against ARF by producing short-chain fatty acids (SCFAs),^[24]^ such as butyrate, whose anti-inflammatory properties can be beneficial for the gut health of piglets during the weaning period.^[25]^ These SCFAs can enhance epithelial barrier integrity and modulate immune responses, mitigating the severity of ARF.^[26]^ Conversely, in CRF, *Ruminococcaceae* UCG005 may promote disease progression through dysregulated SCFA production, leading to systemic inflammation and metabolic imbalances.^[27]^ Additionally, altered gut permeability and microbial translocation can exacerbate kidney damage, contributing to the progression of CRF.^[28]^ Understanding these mechanisms highlights the complex role of the gut microbiota in renal health. Therefore, our findings suggest that *Ruminococcaceae* UCG005 may exert different metabolic functions depending on the stage of kidney disease, potentially through mechanisms involving SCFAs and their interactions with immune cells.^[29]^

Several studies have examined the relationship between the gut microbiota and renal diseases. A recent review by Li et al^[2]^ reported that local oxidative stress and inflammation play pivotal roles in the pathogenesis and progression of CRF and dysbiosis of the gut microbiota. Studies have also concluded that the gut microbiota may serve as potential targets for future therapeutic interventions.^[30]^ Another study reported that propionate and butyrate levels were linked to progressive renal function decline in CRF patients, and early administration of these SCFAs can prevent disease progression in pre-clinical models of acute renal damage, highlighting their therapeutic potential independent of gut microbiota.^[31]^ However, these studies primarily focused on CRF alone or did not comprehensively analyze ARF and CRF simultaneously.

Our research uniquely examined both ARF and CRF, providing a broader understanding of how specific taxa influence different stages of kidney disease. The dual focus allows for more nuanced insights into the temporal dynamics of gut-kidney interactions. For example, the protective effect of *Haemophilus* spp. in ARF suggests a potential early-stage intervention target.^[32]^

One of the key strengths of our study was the simultaneous analysis of ARF and CRF. Previous studies have often concentrated on one condition, limiting their ability to draw comprehensive conclusions about the gut-kidney axis across different disease stages.^[33]^ By integrating the data from both conditions, we can better understand the trajectory of gut microbiota changes as kidney disease progresses.

Moreover, the use of MR, including the IVW approach, strengthens the causal inference of our findings. MR helps to mitigate confounding factors and reverse causality issues, which are common challenges in observational studies.^[34]^

The gut-kidney axis is increasingly being recognized as a critical pathway in renal pathophysiology.^[35]^ Recent studies have shown that gut dysbiosis can lead to systemic inflammation^[36]^ and toxin accumulation, exacerbating kidney damage.^[37]^ Our results support this notion, particularly highlighting the role of *Ruminococcaceae* UCG005 in protecting against ARF and in promoting CRF. This dual role underscores the complexity of gut microbiota-host interactions and suggests that therapeutic strategies targeting specific taxa could be tailored based on disease stage.^[38]^

For instance, therapies aimed at enhancing *Ruminococcaceae* UCG005 levels might benefit patients with ARF, whereas interventions to reduce its abundance could be advantageous for those with CRF. These findings align with emerging evidence from clinical trials investigating probiotics and prebiotics in renal disease management.^[39]^

Despite the robustness of our findings, this study has several limitations. First, the cross-sectional nature of our data limits our ability to establish temporal relationships between changes in gut microbiota and renal disease progression. Longitudinal studies are needed to validate our results and further explore causality. Second, while MR methods help control for confounders, residual confounding factors cannot be entirely ruled out. Third, although substantial, our sample size may still be underpowered to detect small effect sizes.

Nonetheless, our study’s significance lies in its novel approach of simultaneously examining both ARF and CRF, providing valuable insights into the gut-kidney axis. Future research should aim to replicate our findings in larger, more diverse populations and investigate the underlying molecular mechanisms driving the observed associations. Additionally, interventional studies targeting specific gut microbiota taxa could offer new therapeutic avenues for managing renal diseases.^[40]^

## 5. Conclusion

In conclusion, our study provides compelling evidence for distinct gut microbiota profiles associated with both acute and chronic kidney diseases. We identified *Ruminococcaceae* UCG005 as playing opposing roles in ARF and CRF, suggesting its potential as a biomarker and therapeutic target. Our findings highlight the importance of considering the gut-kidney axis in renal disease management and pave the way for personalized treatments based on the gut microbiota composition.

## Acknowledgments

We are grateful to all the participants who participated in this research. We thank the MiBioGen initiative for providing summary statistics from a gut microbiota GWAS. Additionally, we extend our appreciation to major consortia such as FinnGen and the UK Biobank for making GWAS summary data on renal traits available.

## Author contributions

**Conceptualization:** Wenhua Yang.

**Data curation:** Wenhua Yang.

**Formal analysis:** Wenhua Yang.

**Writing – original draft:** Wenhua Yang.

**Writing – review & editing:** Wenhua Yang.
